# Banana Passion Fruit (*Passiflora mollissima* (Kunth) L.H. Bailey): Microencapsulation, Phytochemical Composition and Antioxidant Capacity

**DOI:** 10.3390/molecules22010085

**Published:** 2017-01-17

**Authors:** Almudena García-Ruiz, Amadeo Girones-Vilaplana, Paola León, Diego A. Moreno, Carla M. Stinco, Antonio J. Meléndez-Martínez, Jenny Ruales

**Affiliations:** 1 Department of Food Science and Biotechnology, Escuela Politécnica National, P.O. Box 17-012759, Quito, Ecuador; paito_lt82@hotmail.com (P.L.); jenny.ruales@epn.edu.ec (J.R.); 2 Department of Food Technology, EPSO, University Miguel Hernández, Ctra. Beniel km. 3.2, Orihuela 03312, Alicante, Spain; agvilaplana@gmail.com; 3 Phytochemistry Laboratory, Department of Food Science and Technology, CEBAS-CSIC, Campus de Espinardo, Espinardo, Murcia E-30100, Spain; dmoreno@cebas.csic.es; 4 Food Colour & Quality Laboratory, Department of Nutrition & Food Science, Universidad de Sevilla, Facultad de Farmacia, Sevilla 41012, Spain; cstinco@us.es (C.M.S.); ajmelendez@us.es (A.J.M.-M.)

**Keywords:** banana passion fruit, *Passiflora mollissima*, microencapsulation, flavonoid, carotenoid, antioxidant capacity, proanthocyanidin

## Abstract

*Passiflora mollissima* (Kunth) L.H. Bailey is an exotic fruit native to South America, known as *taxo* in Ecuador. This paper characterizes its flavonoid and carotenoid composition and antioxidant capacity and evaluates the effect of the spray-drying process on its phytochemical composition and antioxidant capacity. A total of 18 flavonoid compounds, nine proanthocyanidins and nine flavan-3-ol monomers, were identified and quantified. Glycosides of (epi)-afzelechin stood out as the most abundant flavonoid. Three carotenoids were identified, with β-carotene having the highest concentration. The DPPH^·^ and ORAC assay methods indicated a high antioxidant capacity. Furthermore, the bioactive content showed a positive and direct correlation with antioxidant capacity. On the other hand, the spray-drying process produced a stable phytochemical composition and antioxidant activity of *taxo*. These results demonstrate the potential applicability of microencapsulated *taxo* as a functional ingredient in the food industry.

## 1. Introduction

Due to their appearance and attractive organoleptic characteristics, novel and interesting fruits recognized as beneficial for human health are leading current food consumption and health-food trends [1,2]. In the context of healthy diets and well-being, South America has a wealth of underexploited native and exotic fruits of great interest for food technology, science and industrial applications, with many nutritional and functional properties yet to be uncovered. 

**Figure 1:**
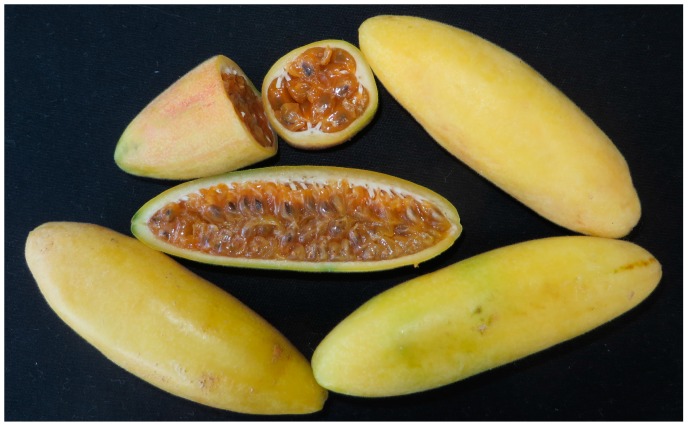
The fruit of banana passion fruit (*Passiflora mollissima* (Kunth) L.H. Bailey).

Banana passion fruit (*Passiflora mollissima* (Kunth) L.H. Bailey, Figure 1), also known as *taxo* in Ecuador, grows in the Andes region at 1800–3600 m.a.s.l. (meters above sea level) in tropical high forests with average temperatures ranging 13–16 °C [3,4]. The oval shaped fruit is yellow and about 8–15 cm long, with a thin but strong skin. The pulp is gelatinous and surrounds small black seeds [5]. *Taxo* is consumed mainly as juice and has an attractive taste much appreciated in the gourmet markets. 

*P. mollissima* is a source of vitamins, calcium, iron, phosphorus, potassium, and fiber [6]. In addition, previous studies on the leaves, pericarps, peel and the edible part of the fruit revealed that *taxo* is rich in natural phenolic antioxidants [4,7,8]. Along these lines, Botero et al. [9] discovered that the antioxidant activity of banana passion fruit can be compared to that of ascorbic acid, the main natural antioxidant used in food industries. On the other hand, the health-promoting effects of *taxo* could be attributed to its polyphenols, specifically flavonoids and carotenoids, which have been strongly associated with antioxidant capacity [10]; additional carotenoid health-promoting activity may stem from other mechanisms, such as pro-oxidant activity and the modulation of cell signalling routes or membrane properties, among others [11,12,13,14,15].

Considering the antioxidant potential and wide range of beneficial biological effects as well as its organoleptic characteristic, banana passion fruit could be employed as a natural additive in the food, pharmaceutical and cosmetic industries. During industrial processing, external factors could affect the quality, stability and functional properties of the bioactive compounds of *P. mollissima.* Industry requires technologies that protect these natural components and the integrity of the organoleptic characteristics. Currently, microencapsulation by spray-drying is the most common technique in the food industry to produce high quality and stable particles, with low cost and flexibility [16]. Moreover, this procedure also enhances bioaccessibility and bioavailability of the functional ingredients [17]. Spray-drying is based on the transformation of a fluid to a solid material, atomizing it in form of tiny drops in a heat-drying medium. The particle size obtained is typically <100 μm in diameter. In this context, the main goal of this study was to investigate the phytochemical composition (flavonoids and carotenoids) and antioxidant capacity of *taxo* and, using microencapsulation, to evaluate banana passion fruit pulp as a source of natural additives for the food industry. 

## 2. Results and Discussion

### 2.1. Enzymatic Hydrolysis

The use of enzymes to obtain pulp increased pulp extraction yield from 60.65% to 75.86% and an increase of soluble solids of 10.5 to 13 °Brix. Since passion fruit juice is 8.52 °Brix [18], banana passion fruit has a sweeter pulp. The water soluble fraction increased due to the liquefaction of the cell walls.

### 2.2. Characterization of Phenolic Compounds 

Complete characterization of phenolic composition requires advanced analytical techniques. Reversed-phase liquid chromatography (RP-LC) is the most common method for the separation and analysis of phenolic compounds while UV-detection and mass spectrometry are the techniques commonly used for identification and quantification of phenolic compounds in fruits [19,20]. HPLC-DAD-ESI/MS^n^ for identification (Table 1) and subsequent HPLC-DAD analysis for quantification (Table 2) were used to characterize the phenolic compounds present in banana passion fruit. 

The retention times (Rt), deprotonated molecular ions ([M − H]^−^), and major fragment ions for the characteristic peaks in the samples are summarized in Table 1. Peak identification was established based on the data in Table 1, using commercial standards when available and previously published data on mass spectra and fragmentations. As shown in Table 1, a total of 18 phenolic compounds were detected, mainly proanthocyanidins (PAs) and flavan-3-ol monomers, all of them corresponding to the flavonoid family. This family of polyphenols was also reported as the major phytoconstituents of another *Passiflora* spp., specifically *P. incarnata* [21].

#### 2.2.1. Proanthocyanidins

Five peaks (peaks 2, 4–6, 8) were detected at *m*/*z* 593 with different elution behavior and were tentatively identified as prodelphinidin dimers. Furthermore, the MS/MS fragmentation ion with *m*/*z* 425 (Table 1) indicated a Retro-Diels-Alder (RDA) fission of the heterocyclic ring, whereas the ion at *m*/*z* 407 was a product of subsequent water elimination [22] and the ion at *m*/*z* 289 indicated an (epi)-catechin unit. The mass spectrum of compound **3** with a [M − H]^−^ ion at *m*/*z* 897 suggested that this compound could be a B-type procyanidin trimer. Peak 7 presented an ion at *m*/*z* 593([M − H]^−^) with the MS analysis in negative mode indicating that this peak contained mixed dimers consisting of two (epi)-catechingallate units and/or (epi)-catechingallate–(epi)-gallocatechin. Peak 9 (Rt = 14.5) exhibited a signal at *m*/*z* 577 suggesting that this compound was a B-type procyanidin dimer. Finally, the mass spectrum of the compound eluting at Rt 30.3 min (Peak 15) exhibited a negative peak at *m*/*z* at 561, which implied it was a propelargonidin dimer composed of one (epi)-catechin and one (epi)-afzelechin unit. 

#### 2.2.2. Flavan-3-ol Monomers

Three compounds (peaks 1, 13 and 18) were identified as (epi)-afzelechin glucoside derivatives based on their UV-Vis and mass spectra data (Table 1). All of them showed a major fragment of ion at *m*/*z* 273 corresponding to (epi)-afzelechin. Peak 10 (Rt = 16.1) showed a [M − H]^−^ ion at *m*/*z* 451. The major MS/MS fragment ion with *m*/*z* 289 (−162 amu) indicated a loss of a hexose from the catechin unit. Glucose is the most common hexose, whereas catechin is the most common flavan-3-ol monomer in nature [23]. In addition, four peaks (11, 14, 16 and 17) displayed a [M − H]^−^ ion at *m*/*z* 435. Its MS/MS analysis revealed the loss of 162 *amu*, indicating the presence of a hexose moiety attached to an (epi)-afzelechin unit (*m*/*z* 273). Finally, a compound at 22.5 min (compound **12**) was tentatively identified as a catechin derivative as suggested from the characteristic deprotonated molecular ion with *m*/*z* 289.

The genus *Passiflora* is known to contain flavonoid *C*-glycosides. In particular, reports on the phenolic profile of *P. incarnata* and *P. edulis*, the most studied species of the genus, showed that these fruits were rich in flavonoids [4,24,25]. These compounds have been detected and identified previously in *taxo* leaves and pericarp [8] and peel and juice [4]. Here, catechin and (epi)-afzelechin were detected as glycosides, which are uncommon in nature [23]. On the other hand, the low degree of proanthocyanidin polymerization detected in *P. mollissima* indicates they may have intestinal bioavailability.

Quantitative analysis of freeze dried fresh fruit indicated that flavan-3-ol monomers were the major constituents of the phenolic fraction (59.4%), with (epi)-afzelechin as the most abundant flavan-3-ol monomer unit (Table 2). The glycosides of (epi)-afzelechin (compounds **1**, **11** and **13**–**18**), 169.7 mg/100 g DW, accounted for approximately 35.4% and 59.7% of the total phenolic and flavan-3-ol monomers, respectively. Biological studies suggested that (epi)-afzelechin may have an anti-inflammatory, anti-oxidant and bone-protective effects [26]. Catechin glycoside content increased when encapsulated (Table 2).

With regard to PAs, (epi)-catechin was the most abundant PA subunit (Table 2). This result is consistent with that described in the literature, which indicates that (epi)-catechin is the predominant constituent unit in most foods containing PAs [27]. The total content of PAs (194.5 mg/100 g), evaluated as the sum of individual PA content, was higher than some fruits described as sources of PAs such as apples (105–126 mg/100 g), similar to plums (216 mg/100 g), but lower than red wine (313 mg/100 g), cocoa (1636 mg/100 g) and cinnamon (8108 mg/100 g) [27,28]. It is important to emphasize that PAs have been linked to many health benefits including antioxidant, anti-inflammatory, antidiabetic, antiallergic, and neuroprotective activities, and reducing the risk of chronic diseases such as cardiovascular diseases and cancers, among others [19,28,29,30]. Prodelphinidin dimers ((epi)-gallocatechin-(epi)-catechin) (compounds **2**, **4**–**6** and **8**)), were the main PAs with 87.9 mg/100 g DW. The PA fraction of banana passion fruit was mainly a mixture of procyanidins and prodelphinidins, also found in other foods such as black currants, barley, grapes, gooseberries, red currants, and wine [28]. Finally, it is important to mention the presence of the propelargonidin dimer (compound **15**) a PA rare in foods [28]. 

Concerning the profile and concentration of the phenolic fraction of microencapsulated banana passion fruit, the results showed that spray-drying did not modify the phenolic profile of *P. mollissima* (Table 2). Most flavonoid compounds in the microencapsulated *taxo* did not exhibit significant differences in concentration with respect to freeze-dried banana passion fruit (Table 2). These results reflect the high stability of these compounds in the spray-drying process. Regarding the flavonoids that were slightly affected by the spray-drying process, the changes observed could be due to the conditions (i.e., temperature) in which the spray-drying process was carried out. This in accordance with the results reported by Susantikarn and Donlao [31] in green tea extracts. Moreover, the spray-drying conditions could also cause changes in phenolic compounds not identified in the study such as flavonols and flavones, among others.

### 2.3. Carotenoids

A previous study by Wondracek et al. [32] demonstrated that the carotenoid profile of passion fruit species showed diversity. Specifically, thirteen carotenoids were detected and identified in *P. edulis*, seven in *P. setacea*, five in *P. cincinnata* and two in *P. nitida*. β-Carotene and antheraxanthin were the carotenoids present in the four species. According to the results presented in Table 3, the carotenoid profile from *P. mollissima* was composed of three major compounds: α- and β-carotene and zeaxanthin. This result contributes to the variability of carotenoids in the *Passiflora* species. From the results reported so far, β-carotene can be considered as the common carotenoid in this genus. In addition, to our knowledge, α-carotene is reported here for the first time as a passion fruit carotenoid. 

Carotenoid variability depends, among many factors, on the maturity of the fruit and the cultivation systems used to grow it [33], as well as the exposure to light, temperature and degree of fruit ripeness, among others [34]. β-Carotene, with 79.74 μg/g, was the main compound of the carotenoid fraction. This concentration is higher than that reported for sweet passion fruit (5.82 μg/g) [35], passion fruit (13.62 μg/g) [36] and other tropical fruit such as arazá pulp (*Eugenia stipitata* McVaugh) (0.44 μg/g) [37]. β-Carotene is the main safe dietary source of vitamin A, essential for normal growth and development, immune system function, and vision [38]. Moreover, β-carotene has antioxidant properties that can decrease the risk of developing chronic degenerative diseases such as cardiovascular disease and cancer [39]. 

In addition, it is important to emphasize the presence of zeaxanthin in *P. mollissima* (Table 3), since there are not many good dietary sources of this carotenoid [40]. This compound together with lutein are two of the most studied carotenoids in terms of health promoting effects because together they comprise the yellow pigment in the macula of the human eye [41]. Furthermore, zeaxanthin has also been associated with a reduction in cataract risk [42] and improved cognitive function in a geriatric population [43]. The zeaxanthin content in banana passion fruit pulp (1.86 μg/g) was higher than the amount reported by Wondracek et al. [32] in *P. setacea* and *P. edulis* (detected but not quantified) and by Garzón et al. [37] in arazá pulp (0.17 μg/g).

Finally, the total content of carotenoid evaluated as the sum of the content of individual pigments was 81.6 μg/g. This amount is within the range reported for several cultivars of *P. edulis* ranging from 15.4 to 251 μg/g [35,36] and higher than that described for other tropical fruits such as arazá pulp (8.06 μg/g) [37], Andean naranjilla fruit pulp (*Solanum quitoense* Lam) (7.94 μg/g) [44] and papaya (*Carica papaya*) (7.93–51.34 μg/g) [45].

Spray drying modification of *P. mollissima* carotenoid concentration was evaluated. As shown in Table 3, the carotenoid content did not show significant differences between banana passion freeze dried fresh fruit and microencapsulated pulp, except for α-carotene. The reasons why the level of α-carotene is higher in the microencapsulated samples are unclear. In general, the carotenoid content of banana passion fruit pulp was not affected by the spray-drying process.

### 2.4. Antioxidant Capacity

The phenolic and carotenoid composition of fruit is associated with its antioxidant properties and involves more than one mechanism. Since there is no official standardized method, antioxidant capacity should be evaluated using various methods [46]. 2,2-Diphenyl-1-picrylhydrazyl (DPPH) and the oxygen radical absorption capacity (ORAC) have been frequently used to estimate antioxidant capacities in fresh fruits and vegetables and their products and foods. The DPPH method is based on the measuring the scavenging activity for DPPH radicals while ORAC assay is based on generation of free radicals using AAPH (2,2-Azobis 2-amidopropane dihydrochloride) and measuring decreasing fluorescence in the presence of free radical scavengers. The results of *taxo* pulp antioxidant capacity are shown in Table 4. The values obtained in the DPPH and ORAC assays (50.1 and 105.2 mM Trolox/100 g, respectively), agree with the range of values reported by other authors [10,47,48,49] and are higher than the antioxidant capacity values reported for other passion fruits [10,24] and other Latin American fruits [10,50] (Table 5). This agrees with previously reported data by Vasco et al. [51] and Contreras-Calderon et al. [6], who have classified *taxo* as a tropical fruit with high antioxidant activity. 

The antioxidant capacity of microencapsulated banana passion fruit and freeze dried fresh fruit did not show significant difference (*p* < 0.05) (Table 4). This agrees with the absence of significant difference in total content of flavonoids and carotenoids for both processing methods (Table 2 and Table 3). The flavonoid and carotenoid content showed a positive and direct correlation with the antioxidant capacity (DPPH and ORAC) (Table 6), especially with ORAC (*r* = 1). A positive and significant correlation has also been obtained by other authors [51,52]. In addition, it is important to emphasize that the concentration of flavonoids was higher than carotenoids (Table 2 and Table 3) which suggest that the high antioxidant capacity observed in *P. mollissima* may be attributed mainly to the polyphenols, and particularly the PAs present in major quantities in the banana passion fruit tested. 

## 3. Experimental Section

### 3.1. Fruit Samples 

Banana passion fruit was purchased at traditional markets in Quito, Ecuador. The fruit was selected, cleaned, washed with tap water and disinfected using 100 ppm of chlorinated water.

### 3.2. Enzymatic Hydrolysis

In order to obtain pulp, an enzymatic hydrolysis treatment was performed. A cocktail of Rapidase Carrots juice (200 ppm/kg banana passion fruit pulp) purchased from Interenzimas Gist-Brocades^®^ (Heerlen, The Netherlands) was used to hydrolyze the pulp. After the hydrolysis for two hours at 30 °C, a refiner separated the pulp from the seeds. The pulp was packed and stored at −20 °C. One fresh fruit fraction was freeze-dried, packed, and stored at 4 °C until analysis. The other fraction was encapsulated with maltodextrins (3%) by a spray dryer in a Minor model Niro Atomizer (Soeborg, Denmark). The process conditions were as follows: inlet temperature of 120 °C and outlet temperature 60–80 °C. A Masterflex (Cole-Palmer, Vermon Hills, IL, USA) peristaltic pump model 77201-60 transported the fluid (pulp and maltodextrins) to the spray dryer at a feed flow of 12 mL/min. The microencapsulated sample was then packed and stored at 4 °C until analysis.

### 3.3. Standards, Chemicals and Solvents

Catechin, β-carotene and zeaxanthin standards were purchased from Sigma-Aldrich Chemie GmbH (Steinheim, Alemania), while α-carotene was isolated from natural sources by a classical chromatographic technique [53]. The reagents 2,2-Diphenyl-1-picrylhidracyl radical (DPPH**·**), 2,2-Azino-bis(3-ethylbenzothiazoline-6-sulphonic acid) diammonium salt (ABTS^+^), 2,2′-Azobis(2-methylpropionamidine) dihydrochloride (AAPH), monobasic sodium phosphate, dibasic sodium phosphate were obtained from Sigma-Aldrich, while 6-Hydroxy-2,5,7,8-tetramethylchroman-2-carboxylic acid (Trolox) was purchased from Fluka Chemika (Neu-Ulm, Switzerland). Meanwhile, formic acid, ethanol, methanol, hexane, acetone, dichloromethane and acetonitrile were all analytical grade (Merck, Darmstadt, Germany). Finally, the chromatographic solvents (HPLC grade-methanol, acetonitrile, ethyl acetate) were also procured from Merck. 

### 3.4. Identification of Phenolic Compounds by HPLC-DAD-ESI/MS^n^ and Quantification by RP-HPLC-DAD

The extraction and analysis of phenolic compounds were performed using the method of Gironés-Vilaplana et al. [50]. Briefly, 0.1 g of lyophilised fruit was extracted with 1.5 mL of 70% MeOH by sonication for 1 h, followed by overnight maceration and other sonication period (1 h). The resulting extract was centrifuged (12,000 rpm, 5 min) and filtered through a 0.22 µm PVDF membrane (Millex-GV syringe filter, Millipore, Bedford, MA, USA). The chromatographic analyses for identification were carried out on a Luna C18 column (250 × 4.6 mm, 5 μm particle size; Phenomenex, Macclesfield, UK). Water/formic acid (99:1, *v*/*v*) and acetonitrile were used as the mobile phases A and B, respectively, with a flow rate of 1 mL/min. The linear gradient started with 8% solvent B, reaching 15% solvent B at 25 min, 22% at 55 min, and 40% at 60 min, which was maintained to 70 min. The injection volume was 20 µL. Identification was carried out by HPLC-DAD-ESI/MS^n^ analyses, using an Agilent HPLC 1100 series model equipped with a photodiode array detector and a mass detector in series (Agilent Technologies, Waldbronn, Germany). The equipment consisted of a binary pump (model G1312A), an autosampler (model G1313A), a degasser (model G1322A), and a photodiode array detector (model G1315B). The HPLC system was controlled by Chem-Station software (Agilent, version 08.03). The mass detector was an ion trap spectrometer (model G2445A) equipped with an electrospray ionization interface, and was controlled by LCMSD software (Agilent, version 4.1). The ionization conditions were 350 °C and 4 kV, for capillary temperature and voltage, respectively. The nebulizer pressure and nitrogen flow rate were 65.0 psi and 11 L/min, respectively. The full-scan mass covered the range of *m*/*z* from 100 to 1200. Collision-induced fragmentation experiments were performed in the ion trap using helium as the collision gas, with voltage ramping cycles from 0.3 to 2 V. The mass spectrometry data were acquired in the negative ionization mode for flavonoids. The MS^n^ was carried out in the automatic mode on the more abundant fragment ion in MS (*n* − 1). For the quantification, the HPLC system (Agilent 1220-Infinity LC) was equipped with a Luna C18 column (25 cm × 0.46 cm, 5 μm particle size; Phenomenex). Flavanols were quantified using catechin as a standard at 280 nm. All the samples were extracted in triplicate and injected three times. The results were expressed as mg/g DW.

### 3.5. Identification and Quantification of Carotenoid Compounds by Rapid Resolution Liquid Chromatography (RRLC) 

Banana passion fruit samples (200 mg) were mixed with 5 mL of methanol/water (60:40, *v*/*v*). Then, the samples were vortexed and sonicated in an ultrasonic bath for 1 min. Afterward, the samples were mixed and vortexed with 5 mL of dichloromethane, and the mixture was vortexed and centrifuged at 18,000× *g* for 3 min. Dichloromethane was carefully removed and the tube dried. Finally, the dry residue was re-dissolved in 50 μL of acetonitrile prior to their injection in the RRLC system. All the samples were extracted in triplicate and injected three times.

The RRLC analyses were made according to Stinco et al. [54] employing an Agilent 1260 system equipped with a diode-array detector, which was set to scan from 200 to 770 nm. The eluents were acetonitrile (solvent A), methanol (solvent B) and ethyl acetate (solvent C). The linear gradient elution was 0 min, 85% A + 15% B; 5 min, 60% A + 20% B + 20% C; 7 min, 60% A + 20% B + 20% C; 9 min, 85% A + 15% B; 12 min, 85% A + 15% B. A C18 Poroshell 120 column (2.7 μm, 5 cm × 4.6 mm) (Agilent, Palo Alto, CA, USA) kept at 28 °C was used as stationary phase. The flow rate was 1 mL/min. Open lab ChemStation software was used and the chromatograms were monitored at 450 nm. Identification was made by comparison of chromatographic and UV-vis spectroscopic characteristics with those of standards. Meanwhile, the quantification was carried out by external calibration from the areas of the chromatographic peaks obtained by DAD detection at 450 nm. The results were expressed as μg/g DW.

### 3.6. Antioxidant Capacity 

Antioxidant activity was determined by the DPPH**∙** and ORAC methods adapted to a microscale. The ability of banana passion fruit samples to scavenge DPPH**∙** radicals was determined according to the method of Mena et al. [55]. The reaction mixture consisted of adding 2 μL of the corresponding diluted sample to the well containing 250 μL of DPPH^∙^ dissolved in methanol. The mixture was then vortexed vigorously and left for 50 min at room temperature in a dark place. The variation in absorbance was measured at 515 nm as the absorption difference between 50 min and 0 min of reaction. 

With regard to the ORAC test, it was performed according to Ou et al. [56]. Briefly, the reaction was carried out at 37 °C in 10 mM phosphate buffer (pH 7.4) and the final assay mixture (200 μL) contained fluorescein (1 μM), 2,2′-azobis(2-methyl-propionamidine)-dihydrochloride (250 mM) and antioxidant (Trolox (10–200 μM) or banana passion fruit samples (at different concentrations)). DPPH**∙** and ORAC assays were performed using 96-well micro plates (Nunc, Roskilde, Denmark) and an Infinite^®^ M200 microplate reader (Tecan, Grödig, Austria). The results were expressed as mmol Trolox/100 g DW.

### 3.7. Statistical Analysis

The data were subjected to analysis of variance (ANOVA) and Pearson’s correlation coefficient, with a 95% confidence level. The software used was Statgraphics Centurion version 16.1.18 (Statgraphics.Net, Madrid, Spain).

## 4. Conclusions

This complete study on the monomeric and oligomeric flavan-3-ols and carotenoid composition of *P. mollissima* (*taxo*) demonstrates that it is a good source of these bioactive compounds. The antioxidant capacity of *taxo* was high and showed a positive and direct correlation with the total flavonoid and carotenoid content. On the other hand, the spray-drying process, in general, does not affect the content of bioactive compounds and antioxidant capacity. This proves that the spray-drying process is a good technology that guarantees the stability of bioactive compounds and functional properties of *taxo*. Overall, the results show that microencapsulated *taxo* could be used as a food ingredient containing bioactive compounds, although more in vivo research and safety evaluations should be done so that appropriate scientifically-based statements and recommendations for dietary intake can be made.

## Figures and Tables

**Table 1:** Tentative identification of phenolics in banana passion fruit pulp samples by HPLC-DAD-ESI/MS^n^.

Peak	Rt (min)	[M − H]^−^	MS/MS Fragments (*m*/*z*)	Proposed Identification
1	6.7	609	441, 435, 273	(*E*)-Afzelechin glucoside derivative
2	7.4	593	425, 407, 289	Prodelphinidin dimer
3	8.0	897	711, 543, 407, 289	Procyanidin trimer (B-type)
4	8.6	593	425, 407, 289	Prodelphinidin dimer
5	9.4	593	425, 407, 289	Prodelphinidin dimer
6	10.4	593	425, 407, 289	Prodelphinidin dimer
7	11.3	881	711, 593, 425, 407, 289	(E)CG-(E)CG or (E)CG-(E)GC
8	13.5	593	425, 289	Prodelphinidin dimer
9	14.5	577	425, 289	Procyanidin dimer B-type (e.g., (E)C-(E)C)
10	16.5	451	289	Catechin glucoside
11	21.3	435	273	(*E*)-Afzelechin glucoside
12	22.5	485	449, 289	Catechin derivative (unidentified)
13	23.8	567	435, 273	(*E*)-Afzelechin glucoside derivative
14	27.8	435	273	(*E*)-Afzelechin glucoside
15	30.3	561	543, 289	Propelargonidin dimer
16	31.9	435	273	(*E*)-Afzelechin glucoside
17	34.8	435	273	(*E*)-Afzelechin glucoside
18	37.1	548	463, 273	(*E*)-Afzelechin glucoside derivative

Rt: retention time. E: epi; C: catechin; G: gallo/gallate.

**Table 2:** Flavonoid composition in freeze dried and microencapsulated banana passion fruit pulp.

Peak	Compounds	Concentration (mg/100 g DW)	
	*Proanthocyanidins*	Freeze Dried	Microencapsulated
2	Prodelphinidin dimer	<LOQ	<LOQ
3	Procyanidin trimer (B-type)	46.2 ^a^ ± 3.8	14.3 ^b^ ± 3.1
4	Prodelphinidin dimer	<LOQ	<LOQ
5	Prodelphinidin dimer	49.1 ^a^ ± 1.1	16.9 ^b^ ± 3.6
6	Prodelphinidin dimer	23.6 ± 2.0	23.9 ± 4.9
7	(E)CG-(E)CG or (E)CG-(E)GC	32.9 ^a^ ± 2.6	22.9 ^b^ ± 0.8
8	Prodelphinidin dimer	15.2 ^b^ ± 0.7	43.4 ^a^ ± 3.3
9	Procyanidin dimer B-type (e.g., (E)C-(E)C)	<LOQ	32.3 ± 1.2
15	Propelargonidin dimer	27.5 ± 4.2	15.9 ± 0.1
	*Σ Proanthocyanidins*	194.5 ± 14.4	169.6 ± 17.0
	***Flavan-3-ol monomers***		
1	(*E*)-Afzelechin glucoside derivative	20.2 ^a^ ± 1.1	7.3 ^b^ ± 1.0
10	Catechin glucoside	91.1 ± 8.4	110.2 ± 1.4
11	(*E*)-Afzelechin glucoside	30.5 ^a^ ± 0.4	22.5 ^b^ ± 0.4
12	Catechin derivative (unidentified)	23.6 ^b^ ± 1.0	29.6 ^a^ ± 0.6
13	(*E*)-Afzelechin glucoside derivative	64.4 ± 2.6	66.9 ± 0.7
14	(*E*)-Afzelechin glucoside	25.9 ± 1.6	24.6 ± 0.0
16	(*E*)-Afzelechin glucoside	22.5 ± 4.8	10.7 ± 1.1
17	(*E*)-Afzelechin glucoside	<LOQ	<LOQ
18	(*E*)-Afzelechin glucoside derivative	6.2 ± 1.2	26.0 ± 7.7
	Σ *Flavan-3-ol monomers*	284.4 ± 21.1	297.8 ± 22.5
	**Σ Flavonoids**	478.9 ± 35.5	467.4 ± 39.5

LOQ: limit of quantification; ^a^, ^b^ Mean values with different letter on the right indicate a statistically significant difference between the two treatments (*p <* 0.05). E: epi; C: catechin; G: gallo/gallate.

**Table 3:** Carotenoid composition in freeze dried and microencapsulated banana passion fruit pulp.

Compounds	Concentration (μg/g DW)	
	Freeze Dried	Microencapsulated
α-carotene	1.64 ^b^ ± 0.50	4.71 ^a^ ± 1.66
β-carotene	79.74 ± 30.38	69.43 ± 12.85
Zeaxanthin	1.86 ± 0.49	1.56 ± 0.36
Σ carotenoids	81.6 ± 31.37	75.7 ± 14.87

^a^, ^b^ Mean values with different letters on the right indicate statistically significant differences between the two treatments (*p <* 0.05).

**Table 4:** Antioxidant capacity in freeze dried and microencapsulated banana passion fruit pulp.

	Antioxidant Capacity (mmol Trolox/100 g DW)	
	Freeze Dried	Microencapsulated
DPPH	50.12 ± 1.78	48.34 ± 1.64
ORAC	105.22 ± 3.05	103.59 ± 4.55

**Table 5:** Antioxidant capacity: *Passiflora* spp. and other Latin American fruits.

Fruit	Antioxidant Capacity (mmol Trolox/100 g DW)	
	DPPH	ORAC
*Passiflora mollissima*	0.09–60.84 [46,47]	0.42–207.85 [10,46,47,48]
*Passiflora* spp.	0.20–6.20 [24]	2.15–8.67 [10]
*Morinda citrifolia*	3.71 [49]	15.08 [49]
*Physalis peruviana*	1.60–4.94 [49]	3.29–24.29 [49]
*Euterpe oleracea*	4.80–6.38 [49]	12.21–43.80 [10,49]
*Carica papaya*	4.41 [10,49]	1.39–8.71 [10,49]

**Table 6:** Pearson’s correlation coefficients (*r*) between bioactive compounds (flavonoids and carotenoids) banana passion fruit pulp (BPFP) and its antioxidant capacity (DPPH and ORAC).

Samples	DPPH	ORAC
Bioactive compounds freeze dried BPFP	0.52	1.00
Bioactive compounds microencapsulated BPFP	0.75	1.00

Significant at *p <* 0.05.

## References

[1] Koolen H.H.F., Silva F.M.A., Gozzo F.C., Souza A.Q.L., Souza A.D.L. (2013). Antioxidant, antimicrobial activities and characterization of phenolic compounds from buriti (*Mauritia flexuosa* L. f.) by UPLC-ESI-MS/MS. Food Res. Int..

[2] Ayala-Zavala J.F., Vega-Vega V., Rosas-Domínguez C., Palafox-Carlos H., Villa-Rodriguez J.A., Wasim Siddiqui M., Dávila-Aviña J.E., González-Aguilar G.A. (2011). Agro-industrial potential of exotic fruit byproducts as a source of food additives. Food Res. Int..

[3] Lobo M., Medina C.I. (2009). Recursos genéticos de pasifloráceas en Colombia. Cultivo, Poscosecha y Comercialización de las Pasifloráceas en Colombia: Maracuyá, Granadilla, Gulupa y Curuba.

[4] Simirgiotis M.J., Schmeda-Hirschmann G., Bórquez J., Kennelly E.J. (2013). The *Passiflora tripartita* (banana passion) fruit: A source of bioactive flavonoid *C*-glycosides isolated by HSCCC and characterized by HPLC-DAD-ESI/MS/MS. Molecules.

[5] Conde-Martínez N., Sinuco D.C., Osorio C. (2014). Chemical studies on curuba (*Passiflora mollissima* (Kunth) L.H. Bailey) fruit flavor. Food Chem..

[6] Leterme P., Buldgen A., Estrada F., Londoño A.M. (2006). Mineral content of tropical fruits and unconventional foods of the Andes and the rain forest of Colombia. Food Chem..

[7] Contreras-Calderón J., Calderón-Jaimes L., Guerra-Hernández E., García-Villanova B. (2011). Antioxidant capacity, phenolic content and vitamin C in pulp, peel and seed from 24 exotic fruits from Colombia. Food Res. Int..

[8] Zucolotto S.M., Fagundes C., Reginatto F.H., Ramos F.A., Castellanos L., Duque C., Schenkel E.P. (2012). Analysis of *C*-glycosyl flavonoids from South American *Passiflora* species by HPLC-DAD and HPLC-MS. Phytochem. Anal..

[9] Botero M.I., Ricaurte S.C., Monsalve C.E., Rojano B. (2007). Capacidad reductora de 15 frutas tropicales. Sci. Tech..

[10] Zapata S., Piedrahita A.M., Rojano B. (2014). Capacidad atrapadora de radicales oxígenos (ORAC) y fenoles totales de frutas y hortalizas de Colombia. Perspect. Nutr. Humana.

[11] Gruszecki W.I., Strzalka K. (2005). Carotenoids as modulators of lipid membrane physical properties. Biochim. Biophys. Acta.

[12] Hughes D.A. (2001). Dietary carotenoids and human immune function. Nutrition.

[13] Mein J.R., Lian F., Wang X.D. (2008). Biological activity of lycopene metabolites: Implications for cancer prevention. Nutr. Rev..

[14] Palozza P. (2004). Carotenoids and modulation of cancer: Molecular targets. Curr. Pharmacogenom..

[15] Sharoni Y., Linnewiel-Hermoni K., Khanin M., Salman H., Veprik A., Danilenko M., Levy J. (2012). Carotenoids and apocarotenoids in cellular signaling related to cancer: A review. Mol. Nutr. Food Res..

[16] Munin A., Edwards-Lévy F. (2011). Encapsulation of natural polyphenolic compounds; A review. Pharmaceutics.

[17] Desai K., Park H. (2005). Recent development in microencapsulation of foods ingredients. Dry. Technol..

[18] Vera Calle E., Ruales J., Dornier M., Sandeauxc J., Sandeauxc R., Pourcelly G. (2002). Deacidification of the clarified passion fruit juice (*P. edulis* f. *flavicarpa*). Desalination.

[19] Navarro M., Núñez O. (2015). Liquid Chromatography-Mass Spectrometry in the analysis and characterization of proanthocyanidins in natural products. Proanthocyanidins: Food Sources, Antioxidant Properties and Health Benefits.

[20] Lin L.-Z., Sun J., Chen P., Monagas M.J., Harnly J.M. (2014). UHPLC-PDA-ESI/HRMS^n^ profiling method to identify and quantify oligomeric proanthocyanidins in plant products. J. Agric. Food Chem..

[21] Dhawan K., Dhawan S., Sharma A. (2004). *Passiflora*: A review update. J. Ethnopharmacol..

[22] Friedrich W., Eberhardt A., Galensa R. (2000). Investigation of proanthocyanidins by HPLC with electrospray ionization mass spectrometry. Eur. Food Res. Technol..

[23] Ojwang L.O., Yang L., Dykes L., Awika J. (2013). Proanthocyanidin profile of cowpea (*Vigna unguiculata*) reveals catechin-*O*-glucoside as the dominant compound. Food Chem..

[24] Bendini A., Cerretani L., Pizzolante L., Gallina-Toschi T., Guzzo F., Ceoldo S., Marconi A.M., Andreetta F., Levi M. (2006). Phenol content related to antioxidant and antimicrobial activities of *Passiflora* spp. extracts. Eur. Food Res. Technol..

[25] Pierson J.T., Dietzgen R.G., Shaw P.N., Roberts-Thomson S.J., Monteith G.R., Gidley M.J. (2012). Major Australian tropical fruits biodiversity: Bioactive compounds and their bioactivities. Mol. Nutr. Food Res..

[26] Wong K.C., Law M.C., Wong M.S., Chan T.H. (2014). Development of a UPLC-MS/MS bioanalytical method for the pharmacokinetic study of (−)-epiafzelechin, a flavan-3-ol with osteoprotective activity, in C57BL/6J mice. J. Chromatogr. B.

[27] Gu L., Kelm M.A., Hammerstone J.F., Beecher G., Holden J., Haytowitz D., Prior R.L. (2003). Screening of foods containing proanthocyanidins and their structural characterization using LC-MS/MS and thiolytic degradation. J. Agric. Food Chem..

[28] Prior R.L., Gu L. (2005). Occurrence and biological significance of proanthocyanidins in the American diet. Phytochemistry.

[29] Neilson A.P., O’Keefe S.F., Bolling B.W. (2016). High-Molecular-Weight proanthocyanidins in foods: Overcoming analytical challenges in pursuit of novel dietary bioactive components. Annu. Rev. Food Sci. Technol..

[30] Santos-Buelga C., Scalbert A. (2000). Proanthocyanidins and tannin-like compounds—Nature, occurrence, dietary intake and effects on nutrition and health. J. Sci. Food Agric..

[31] Susantikarn P., Donlao N. (2016). Optimization of green tea extracts spray drying as affected by temperature and maltodextrin content. Int. Food Res. J..

[32] Wondracek D.C., Faleiro F.G., Sano S.M., Vieira R.F., Agostini-Costa T.S. (2011). Composição de carotenoides em passifloras do Cerrado. Rev. Bras. Frutic..

[33] Rotili M.C.C., Vorpagel J.A., Braga G.C., Kuhn O.J., Salibe A.B. (2013). Antioxidant activity, chemical composition and conservation of yellow passion fruit packed with PVC film. Rev. Bras. Frutic..

[34] Borguini R.G., Bastos D.H.M., Moita-Neto J.M., Capasso F.S., Torres E.A.F.S. (2013). Antioxidant potential of tomatoes cultivated in organic and conventional systems. Braz. Arch. Biol. Technol..

[35] Pertuzatti P.B., Sganzerla M., Jacques A.C., Barcia M.T., Zambiazi R.C. (2015). Carotenoids, tocopherols and ascorbic acid content in yellow passion fruit (*Passiflora edulis*) grown under different cultivation systems. LWT Food Sci. Technol..

[36] Da Silva S.R., Mercadante A.Z. (2002). Composição de carotenóides de maracujá-amarelo (*Passiflora edulis* flavicarpa) in natura. Ciênc. Tecnol. Aliment..

[37] Garzón G.A., Narváez-Cuenca C.E., Kopec R.E., Barry A.M., Riedl K.M., Schwartz S.J. (2012). Determination of carotenoids, total phenolic content, and antioxidant activity of arazá (*Eugenia stipitata* McVaugh), an Amazonian fruit. J. Agric. Food Chem..

[38] Institute of Medicine, Food and Nutrition Board (2000). Beta-carotene and other carotenoids. Dietary Reference Intakes for Vitamin C, Vitamin E, Selenium, and Carotenoids.

[39] Pavia S.A., Russell R.M. (1999). β-carotene and other carotenoids as antioxidants. J. Am. Coll. Nutr..

[40] Murillo E., Meléndez-Martínez A.J., Portugal F. (2010). Screening of vegetables and fruits from Panama for rich sources of lutein and zeaxanthin. Food Chem..

[41] Bone R.A., Landrum J.T., Fernandez L., Tarsist S.L. (1988). Analysis of the macular pigment by HPLC: Retinal distribution and age study. Investig. Ophthalmol. Vis. Sci..

[42] Moeller S.M., Jacques P.F., Blumberg J.B. (2000). The potential role of dietary xanthophylls in cataract and age-related macular degeneration. J. Am. Coll. Nutr..

[43] Johnson E.J., Vishwanathan R., Schalch W., Poon L.W., Wittwer J., Johnson M.A., Hausman D., Davey A., Green R.C., Gearing M. (2011). Brain levels of lutein (L) and zeaxanthin (Z) are related to cognitive function in centenarians. FASEB J..

[44] Gancel A.-L., Alter P., Dhuique-Mayer C., Ruales J., Vaillant F. (2008). Identifying carotenoids and phenolic compounds in naranjilla (*Solanum quitoense* Lam. Var. Puyo Hybrid), an Andean fruit. J. Agric. Food Chem..

[45] Wall M.M. (2006). Ascorbic acid, vitamin A, and mineral composition of banana (*Musa* sp.) and papaya (*Carica papaya*) cultivars grown in Hawaii. J. Food Compos. Anal..

[46] Frankel E.N., Meyer A.S. (2000). The problems of using one-dimensional methods to evaluate multifunctional food and biological antioxidants. J. Agric. Food Chem..

[47] Gil M., Restrepo A., Millán L., Alzate L., Rojano B. (2014). Microencapsulation of banana passion fruit (*Passiflora tripartita* var. Mollissima): A new alternative as a natural additive as antioxidant. Food Nutr. Sci..

[48] Chaparro R.D.C., Maldonado C.M.E., Urango M.L.A., Rojano B.A. (2015). Propiedades quimiopreventivas de *Passiflora mollissima* (Kunth) L.H. Bailey (curuba larga) contra cáncer colorrectal. Rev. Cuba. Plantas Med..

[49] Rojano B.A., Zapata K., Cortes F.B. (2012). Capacidad atrapadora de radicales libres de *Passiflora mollissima* (Kunth) L.H. Bailey (curuba). Rev Cuba. Plantas Med..

[50] Gironés-Vilaplana A., Baenas N., Villaño D., Speisky H., García-Viguera C., Moreno D.A. (2014). Evaluation of Latin-American fruits rich in phytochemicals with biological effects. J. Funct. Foods.

[51] Vasco C., Ruales J., Kamai-Eidin A. (2008). Total phenolic compounds and antioxidant capacities of major fruits from Ecuador. Food Chem..

[52] Almeida M.M.B., de Sousa P.H.M., Arriaga Â.M.C., do Prado G.M., Magalhães C.E.D.C., Maia G.A., de Lemos T.L.G. (2011). Bioactive compounds and antioxidant activity of fresh exotic fruits from northeastern Brazil. Food Res. Int..

[53] Meléndez-Martínez A.J., Britton G., Vicario I.M., Heredia F.J. (2006). Relationship between the colour and the chemical structure of carotenoid pigments. Food Chem..

[54] Stinco C.M., Benítez-González A.M., Hernanz D., Vicario I.M., Meléndez-Martínez A.J. (2014). Development and validation of a rapid resolution liquid chromatography method for the screening of dietary plant isoprenoids: Carotenoids, tocopherols and chlorophylls. J. Chromatogr. A.

[55] Mena P., García-Viguera C., Navarro-Rico J., Moreno D.A., Bartual J., Saura D., Martí N. (2011). Phytochemical characterisation for industrial use of pomegranate (*Punica granatum* L.) cultivars grown in Spain. J. Sci. Food Agric..

[56] Ou B., Hampsch-Woodill M., Prior R.L. (2001). Development and validation of an improved oxygen radical absorbance capacity assay using fluorescein as the fluorescent probe. J. Agric. Food Chem..

